# Supplementary material for: Identification of photoperiod-induced specific miRNAs in the adrenal glands of Sunite sheep (Ovis aries)
Source: Front Vet Sci. 2022 Jul 22;9:888207. doi: 10.3389/fvets.2022.888207 (PMC9354845; doi:10.3389/fvets.2022.888207)
Supplement: Supplementary file 1 [file Data_Sheet_1.ZIP › Supplementary file/miRDeep2 file.zip!/miRDeep2 file/Novel_Structure/SP42A3/Novel_476.pdf]

Provisional ID	:	NC_019468.2_17379
Score total	:	3.8
Score for star read(s)	:	3.9
Score for read counts	:	-3.3
Score for mfe	:	1.7
Score for randfold	:	1.6
Score for cons. seed	:	
Total read count	:	5
Mature read count	:	2
Loop read count	:	0
Star read count	:	3

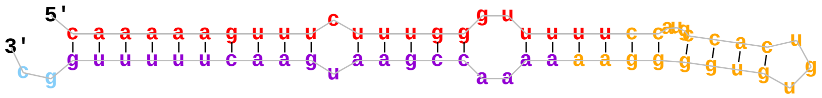

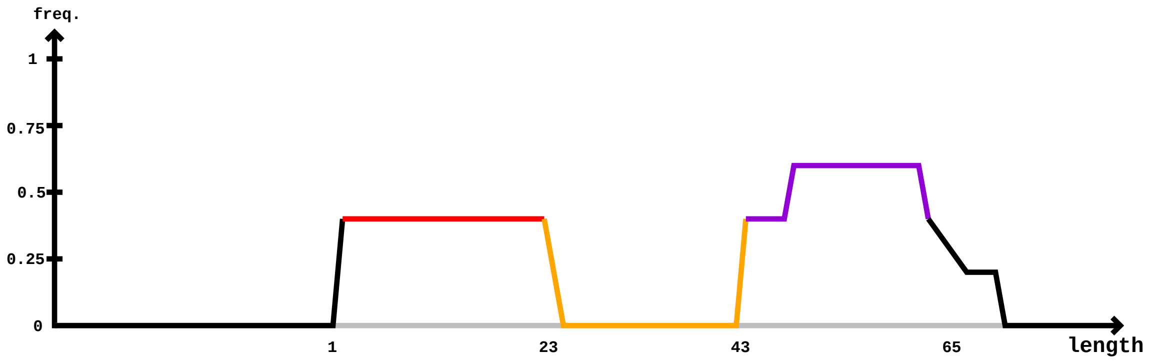

Mature Star

```
5'- caguggaaguaauuuguauuggguugaccaaaaaguuucuuuggguuuuuccaugccacugugugggggaaaaaaccgaaugaacuuuuugccaacacaauauuuucuu  -3'  obs
    caguggaaguaauuuguauuggguugaccaaaaaguuucuuuggguuuuuccaugccacugugugggggaaaaaaccgaaugaacuuuuuggccaacacaauauuuucuu       exp
    ....(((((......((((((.((((.(((((((((((.(((((..((((((...((((...))))))))))..))))).)))))))))))).)))).)))))))))))).  reads    mm    sample
    ............................caaaaaguuuGuuuggguuuuu...........................................................  2        1     seq
    ......................................................................aaaaccgaaugaacuuuuu....................  1        0     seq
    ......................................................................aaaaccgaaugaacuuuuug...................  1        0     seq
    ............................................................................Ggaaugaacuuuuuggccaaca...........  1        1     seq
```
